# Spatiotemporal Variation of *Osmanthus fragrans* Phenology in China in Response to Climate Change From 1973 to 1996

**DOI:** 10.3389/fpls.2021.716071

**Published:** 2022-01-20

**Authors:** Xianping Wang, Yinzhan Liu, Xin Li, Shibin He, Mingxing Zhong, Fude Shang

**Affiliations:** ^1^Key Laboratory of Plant Stress Biology, School of Life Sciences, Henan University, Kaifeng, China; ^2^School of Software Engineering, Taiyuan University of Technology, Taiyuan, China; ^3^Tourism College, Xinyang Normal University, Xinyang, China; ^4^College of Life Sciences, Henan Agricultural University, Zhengzhou, China; ^5^Henan Engineering Research Center for Osmanthus Germplasm Innovation and Resource Utilization, Henan Agricultural University, Zhengzhou, China

**Keywords:** climate change, spring leaf phenology, autumn flowering phenology, *Osmanthus fragrans*, China

## Abstract

Climate change greatly affects spring and autumn plant phenology around the world consequently, and significantly impacts ecosystem function and the social economy. However, autumn plant phenology, especially autumn flowering phenology, has not been studied so far. In this study, we examined the spatiotemporal pattern of *Osmanthus fragrans* phenology, including both leaf phenology (the date of bud-bust, BBD; first leaf unfolding, FLD; and 50% of leaf unfolding, 50 LD) and flowering phenology (the date of first flowering, FFD; peak of flowering, PFD; and end of flowering, EFD). Stepwise multiple linear regressions were employed to analyze the relationships between phenophases and climatic factors in the long term phenological data collected by the Chinese Phenological Observation Network from 1973 to 1996. The results showed that spring leaf phenophases and autumn flowering phenophases were strongly affected by latitude. BBD, FLD, and 50LD of *O. fragrans* were delayed by 3.98, 3.93, and 4.40 days as per degree of latitude increased, while FFD, PFD and EFD in *O. fragrans* advanced 3.11, 3.26, and 2.99 days, respectively. During the entire study period, BBD was significantly delayed across the region, whereas no significant trends were observed either in FLD or 50LD. Notably, all flowering phenophases of *O. fragrans* were delayed. Both leaf and flowering phenophases negatively correlated with growing degree-days (GDD) and cold degree-days (CDD), respectively. BBD and FLD were negatively correlated with total annual precipitation. In addition to the effects of climate on autumn flowering phenology, we found that earlier spring leaf phenophases led to delayed autumn flowering phenophases. Our results suggest that future climate change and global warming might delay the phenological sequence of *O. fragrans.* Our findings also advanced the flowering mechanism study of autumn flowering plants, and facilitated the accurate prediction of future phenology and climate change.

## Introduction

Phenology is the study of the timing of recurring life-cycle events in plants, which rely on various biotic and abiotic factors (Lieth, 1974) and are triggered by changes in environmental conditions (Cong et al., 2017; Liu et al., 2021). Plant phenology is one of the most reliable biological indicators of climate change (Fu et al., 2017), and changes in plant phenology have important impacts on ecosystem structure and function (Fu et al., 2020), including carbon, water and nutrient cycling, hydrology, demography and biological interactions (Estiarte and Peñuelas, 2015; Xie et al., 2018). These changes can also cause a feedback loop that further augmenting changes in the climate system (Thackeray et al., 2016; Zeng et al., 2017). Monitoring plant phenological processes is important to improve our understanding of the impacts of global warming on plant and ecosystem function (Richardson et al., 2013; Fu et al., 2017; Chen et al., 2019). Previous studies have mainly focused on spring phenology, but less on autumn phenology (Gallinat et al., 2015), which also controls nitrogen cycling, ecosystem functions, and the associated feedbacks to climate systems (Visser, 2016). Moreover, the delayed autumn phenology may have greater influences than advanced spring phenology (Liu et al., 2016a), for instance in the regulation of carbon balance in temperate plants (Richardson et al., 2009). Therefore, studying the change of phenological sequence and the corresponding climatic drivers can improve our understanding of the response of species-specific phenology to environmental factors, as well as the responses of plants and ecosystem to ongoing climate change.

The magnitude of plant phenological responses to climate change is diverse and varies significantly in terms of geographical location, time and species (Chen et al., 2017). According to the Hopkins’ Bioclimatic Law, increased latitude and altitude can lead to delayed spring phenology, advanced autumn phenology, and an overall shorter growing season (Wang et al., 2015; Zhu et al., 2018). Many previous studies have investigated the timing of phenological events and their connection to climate change by using long-term records and remote sensing data in temperate and cold regions. Earlier spring phenology and later autumn phenology have been confirmed by these studies (Jeong et al., 2011; Gerst et al., 2016). However, in contrast to the wide attention on the variation of spring phenology, autumn flowering phenology has not received as much attention, because of the difficulties in interpretation (Xie et al., 2018). As a result, there are only a few studies that have investigated the dynamics of autumn phenology and the related controlling factors (Estiarte and Peñuelas, 2015; Liu et al., 2016b), particularly at regional scales (Gallinat et al., 2015; Fu et al., 2018). Shifting flowering phenology is a key biological indicator of climate change. However, the response of autumn flowering species to climate change is unclear. More studies are needed on autumn phenology and its controlling factors to gain a more holistic understanding of the impact of global climate change on ecological processes (Peng et al., 2019).

Temperature, precipitation and photoperiod are considered the primary controls of phenology (Fu et al., 2020). Among which temperature is the most important indicators (Li et al., 2018; Cheng et al., 2021). Warming advances spring plant phenology in temperate and boreal zones (Richardson et al., 2010). In subtropical regions, increase in chilling can advance the leaf-out (Song et al., 2020a). Nonlinear relationships (Cook et al., 2012) have been reported between spring phenology and temperature (Fu et al., 2015). The response of plant spring phenology to precipitation was inconclusive (Hänel and Tielbörger, 2015). Higher preseason precipitation could also alter spring phenology (Dai et al., 2013; Piao et al., 2019; Zhou et al., 2019), because more precipitation may increase the heat demand of spring phenology (Fu et al., 2014). Photoperiod may also influence bud burst, but generally in a minor way (Chen et al., 2017; An et al., 2020). To date, the mechanisms by which photoperiod affects phenology remain unexplored experimentally (Liu et al., 2016a). Compared to spring phenology, the linkages between autumn phenology and climatic factors have not been better identified (Fu et al., 2017; Piao et al., 2019; An et al., 2020). A higher preseason temperature delays autumn phenology in temperate regions (Cong et al., 2017; Zhu et al., 2017; Li et al., 2018; Fu et al., 2020). Photoperiod (Way and Montgomery, 2015), drought and heavy precipitation (Xie et al., 2018), precipitation (Richardson et al., 2013; Estiarte and Peñuelas, 2015) have been considered to influence autumn phenology. The drivers influencing autumn phenology vary across regions and among plant species. Low temperature and short daylength are considered as the dominant cues for autumn phenology (Gunderson et al., 2012). At high latitude colder regions, leaf senescence may be more responsive to photoperiod rather than temperature (Way and Montgomery, 2015). In contrast, temperature is a key factor for autumn leaf color (Tanino et al., 2010). In addition, previous studies have indicated that spring phenology also affects autumn phenology (Fu et al., 2014, 2018; Liu et al., 2016b), where an earlier spring phenology leads to a later autumn phenology (Fu et al., 2018; Škrk et al., 2020). The mechanism between these climatic factors and phenology on large spatial and temporal scales is still unclear.

In this study, we estimate the spatiotemporal variations of spring leaf phenology and autumn-flowering phenology of *Osmanthus fragrans* in response to climate change in China from 1973 to 1996. Specifically, this study attempted to solve the following questions: (i) What are the spatiotemporal variations of the spring and autumn phenology of *O. fragrans* in China from 1973 to 1996; (ii) How do the spatiotemporal variations in the spring leaf and autumn flowering phenology of *O. fragrans* correlate with climate factors? and (iii) What is the relationship between spring leaf phenology and autumn flowering phenology? In view of about *O. fragrans* was an autumn flowering species, according to the results of Sherry et al. (2007), we hypothesize that *O. fragrans* will flower earlier at high latitude compared to low latitude because of the variation of temperature. In addition, considering the spatial variation of precipitation and photoperiod is disordered, we hypothesize that photoperiod did not regulate the variation of flowering time of *O. fragrans*.

## Materials and Methods

### Study Area and Plant Species

**FIGURE 1:**
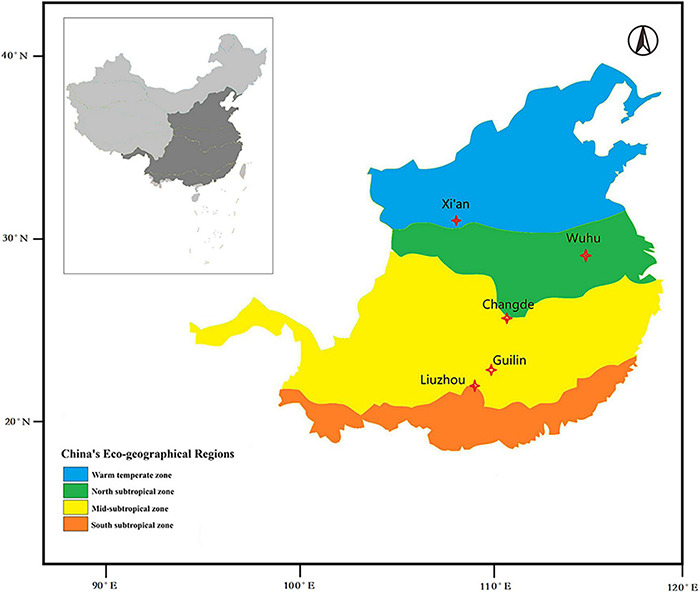
Locations of observation sites, overlain on a map of China’s eco-geographical regions (issued by the Institute of Geographical Sciences and Natural Resources Research, Chinese Academy of Sciences). The five study locations represent three different climatic regions for *O. fragrans* cultivation.

**FIGURE 2:**
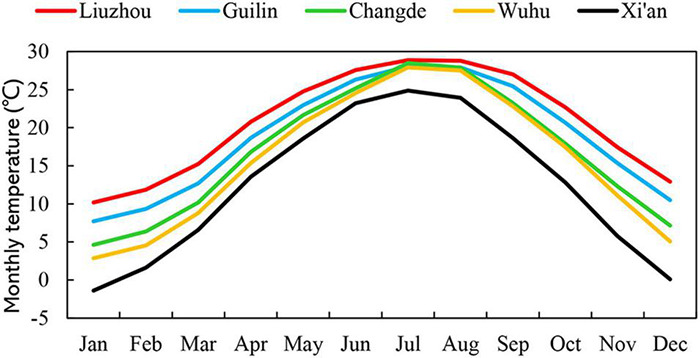
Mean monthly temperatures at the five observation sites.

The study area extended from 24 to 34°N and from 108 to 118°E, and the altitude ranged from 23 to 720 m. This provided a broad geographical coverage of the cultivation distribution area of *O. fragrans*. Observations centered on five stations in three different climatic zones of China. According to China’s eco-regional classification, one site (Xi’an) was in the warm temperate zone. Another (Wuhu) was in the northern subtropical zone. One station (Changde) was located in the transition zone from the north subtropical zone to the mid-subtropical zone, and two sites (Guilin and Liuzhou) were in the mid-subtropical zone (Figure 1). The temperature regimes of these five sites are shown in Figure 2 and Supplementary Data 1.

*Osmanthus fragrans* belongs to the Oleaceae family. It is an evergreen tree or shrub that originated in southwest China. This species flowers in the autumn and is grown ornamentally for its attractive foliage and fragrant edible flowers. China is the distribution center of *O. fragrans* and is rich in germplasm resources for the species, which is a model species of the Oleaceae family. It is one of the top ten most famous flowers, having been cultivated in China for over 2,500 years. Now it is widely distributed, has been planted in open fields from Shandong (36° north latitude) to San’ya (18° north latitude), Hainan Province. *O. fragrans* is an excellent garden tree and an important spice plant. As ornamental plants, their flowering seasons have important aesthetic and economic benefits for some regions. For example, *O. fragrans* festivals are of major cultural importance in certain parts of China.

### Phenological Data and Climate Data

The phenological data of *O. fragrans* collected at five locations in the study area from 1973 to 1996 was obtained from the Chinese Phenological Observation Network (CPON) (Supplementary Data 2). Details of the phenological observation method conform to standardized observation criteria and guidelines (Wan and Liu, 1979; Dai et al., 2014). Six phenophases were investigated, including three spring leaf phenophases and three autumn flowering phenophases. The BBD was defined as the date the appearance of the first green leaf tip on a few twigs of the observed tree (Murray et al., 1989; Basler and Körner, 2014). The FLD was defined as the date when the first batch of leaves is fully spread on a few twigs of the observed tree; whereas 50LD is defined as the date when leaves are fully spread on 50% of twigs of the observed tree (Chen et al., 2017). The FLD represented the date when the first leaf unfolded in at least one individual, while the 50LD represented the date when more than 50% of individuals had unfolded their leaves (Huang et al., 2019). The FFD was defined as the date when 10% flowers were open, the PFD was defined as the date when 90% flowers were in full bloom, and the EFD was the date on which the majority of small flowers (about 90%) had withered or dropped (Yang, 2013). To ensure the accuracy and validity of the statistical analysis, the phenological time series with more than 10 years’ worth of observations were chosen (Dai et al., 2014). The original phenological data was preprocessed according to the 30-day rule to remove outliers that possibly had been recorded incorrectly (Wang et al., 2018). Only five stations (Liuzhou, Guilin, Changde, Wuhu, and Xi’an) in the time period, from 1973 to 1996, were chosen for analysis. In these stations, *O. fragrans* was planted in batches and long observed. Generally, more than 5 trees of the same species were selected as the observation target to record the phenology, and a branch without shade was selected from the middle and upper four directions (East, South, West, and North) of each tree to observe its phenology. In total, 70, 80, 82, 77, 72, and 76 time series were analyzed for BBD, FLD, 50LD, FFD, PFD, and EFD, respectively. All onset data was converted to Julian day (DOY). The number of phenological samples (the total number of observations) observed at each site did differ (Table 1 and Supplementary Data 3). All phenological data were obtained from cultivated populations of *O. fragrans.*

**TABLE 1:** Number of years of phenological period during 1973–1996 years at each location.

Site	Eco-geographical region	Location coordinates	BBD(n)	FLD(n)	50LD(n)	FFD(n)	PFD(n)	EFD(n)
Liuzhou	Mid-subtropical zone	24°21′N, 109°24′E	12	12	13	12	12	12
Guilin	Mid-subtropical zone	25°20′N, 110°18′E	15	18	19	17	17	19
Changde	Transition zone	28°95′N, 111°40′E	16	16	17	14	15	14
Wuhu	Northern subtropical zone	31°42′N, 118°31′E	11	14	14	14	14	13
Xi’an	Warm temperate zone	34°56′N, 108°59′E	16	20	19	18	14	18

*BBD, date of bud-burst; FLD, date of first leaf unfolding; 50LD, date of 50% of leaf unfolding; FFD, date of first flowering, PFD, date of peak flowering and EFD, end of flowering day.*

The data of daily climate factors, including daily mean air temperature, daily precipitation and daily sunshine hours were obtained from the China Meteorological Sharing Service Network. The meteorological stations are all located at or nearby the corresponding phenological stations. They were Liuzhou and Guilin stations in Guangxi Autonomous Region, Changde station in Hunan Province, and Wuhu station in Anhui Province, respectively. However, because we cannot directly download meteorological data of Xi’an in the China Meteorological Sharing Service Network, the data were collected from the Great Wild Goose Pagoda station in Xi’an Meteorological Bureau. These data are well checked and normalized by China Meteorological Administration before being issued.^1^

Growing degree-days (GDD) is a heat accumulation index for analyzing the effect of temperature on spring phenophases (Fu et al., 2014; Liu et al., 2019). Cold degree-days (CDD) were applied as a corresponding metric to explain the variation in autumn phenology (Dragoni and Rahman, 2012; Xie et al., 2018).

According to McMaster and Wilhelm (1997), GDD was calculated as the thermal sum of the difference between daily mean temperature (T_*m*_) and the base temperature (T_*b*_) between DOY_1_ and DOY_2_ [formula (1)].


(1) GDD=∑DOY1DOY2((Tm-Tb)whenTm>Tb 0whenTm≤Tb)

Where, *DOY*_1_ represents the starting date of the research phase. It was set as January 1st here. *DOY*_2_ represents the ending date of the research phase. To analyze the correlation between spring phenology and GDD across different stations and years, the end dates of GDD were set to April 30th for BBD, FLD, and 50LD (Zhu et al., 2018).

Temperatures above 0°C (Hänninen, 1990) and a temperature of 5°C (Marchin et al., 2015) represented the minimum temperature threshold required for stimulating budburst. Experiments have shown that base temperature is generally accepted to be below 10°C, although it is species-specific (Harrington et al., 2010; Malyshev, 2020). Three base temperatures, 0, 5, and 10°C were tested in this study. Hereafter we only report results using the base temperature of 5°C. The temperature threshold of 0°C and 10°C were found similar results (Supplementary Table 1 and Supplementary Data 4).

Cold Degree-Days was calculated by summing the deviation between the base temperature (T_*b*_) and daily mean temperature (T_*m*_) between DOY_1_ and DOY_2_ [formula (2)].


(2) CDD=∑DOY1DOY2((Tb-Tm)whenTb>Tm 0whenTb≤Tm)

The length of preseason was chosen to be 2 months before mean phenological events following previous studies (Cong et al., 2013; Sakkir et al., 2015). Temperatures during the preseason is the most important for flowering phenology for most species in Europe and Asia (Fu et al., 2015; Zhang et al., 2015). In the previous researches, DOY_1_ was set as 1st August, DOY_2_ was set to 31st October (Xie et al., 2018; Zhu et al., 2018). The earliest flowering phenology of *O. fragrans* occurs in Xi’an was in late September, and the average flowering phenology was in the early October across all the stations and years. In this manuscript the DOY_1_ was set as 1st July, DOY_2_ was set to 31st October to calculate the CDD.

The threshold temperature is accepted to be between 20 and 26°C (Delpierre et al., 2009). The base temperature for CDD is 25°C in this paper. We also tested the base temperature threshold of 20 and 30°C, and very similar results were observed (Supplementary Table 2 and Supplementary Data 5).

The cumulative precipitation (PPT) and cumulative sunshine duration (SSD) during the same periods of GDD or CDD were calculated for each station and each year. Considering photoperiod may also regulate phenology, we took the relationships between daily sunshine hours (i.e., day length) and phenological variables as the photoperiod effect.

### Statistical Analyses

A stepwise multiple linear regression model was used to estimate the effect of geographical factors and years on the spatiotemporal variation of phenology in the five study areas from 1973 to 1996. Geographical factors (longitude, latitude, and altitude) and year were used as independent variables, and phenological metrics were dependent variable. The variance inflation factor (VIF) was used to quantify the multi-collinearity among variables in the models. The model was accepted when the VIF of individual predictors was less than three, which indicated a lack of multi-issues with collinearity (Zuur et al., 2010). Squared semi-partial correlation coefficients were used to test the relative contribution of each independent variable in a given model, which were determined as the reduction in R^2^ with removing a given predictor from the set of independent variables (Watson et al., 2011). Values were reported as fractions of the original R^2^ (Zhu et al., 2018).

Stepwise multiple linear regression analysis was used to analyze the climate controls on the phenophases across all stations and years. Simple linear regression analyses were conducted between climatic factors and phenophases to quantify the effects of climatic factors on the phenophases, One-way ANOVA was used to test the significance of climate factors including GDD, CDD, TTP, and SSD in five stations. The correlation between spring and autumn phenophases was assessed using simple linear correlation. *P*-values less than 0.05 were considered significant. All analyses were performed using SPASS 22.0 (SPASS Inc., Chicago, IL, United States).

## Results

### Spatiotemporal Variation of Spring Leaf Phenophases in *Osmanthus fragrans*

**FIGURE 3:**
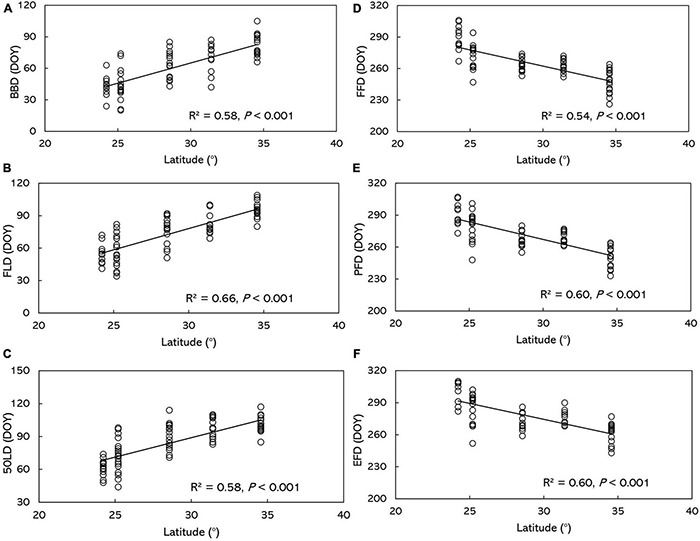
Relationship between *O. fragrans* phenophase and latitude. **(A)** BBD, **(B)** FLD, **(C)** 50LD, **(D)** FFD, **(E)** PFD, and **(F)** EFD. BBD, date of bud-burst; FLD, date of first leaf unfolding; 50LD, date of 50% of leaf unfolding; FFD, first flowering day; PFD, peak flowering day; EFD, end of flowering day. DOY indicates the Julian day of year.

Based on the results from the multiple stepwise regression analysis, the phenophases showed significant correlation with geographical factors (Table 2 and Supplementary Data 6). Latitude has the largest squared semi-partial correlation coefficients of all the factors, suggesting that it was the most profound factor that influencing phenology, moreover, it also was the factor that contributes most to the spatial variation in all phenological metrics. Due to the strong dependence of phenophases on latitude, BBD, FLD and 50LD showed significant positive correlations with latitude in *O. fragran*s (*P* < 0.001) (Figures 3A–C and Supplementary Data 7).

**TABLE 2:** Stepwise multiple linear regression coefficients of each independent variable related to phenological metrics.

Phenological phases	Latitude (days °–1)	Longitude (days °–1)	Altitude (days m–1)	Year (days yr–1)	N	R^2^	F
BBD	3.98 (0.63)***	−0.05 (0.01)	−0.03 (0.00)	0.69 (0.11)**	70	0.62***	58.04
FLD	3.93 (0.66)***	0.00 (0.00)	−0.06 (0.00)	0.08 (0.02)	80	0.66***	152.00
50 LD	4.40 (0.57)***	−0.18 (0.02)	−0.018 (0.10)**	0.11 (0.03)	82	0.61***	65.38
FFD	−3.11 (0.59)***	0.05 (0.01)	−0.06 (0.00)	0.67 (0.15)***	77	0.61***	59.80
PFD	−3.26 (0.60)***	0.13 (0.04)	−0.08 (0.01)	0.52 (0.10)**	72	0.61***	55.78
EFD	−2.99 (0.55)***	0.10 (0.02)	−0.02 (0.00)	0.43 (0.06)*	76	0.55***	46.44

BBD, date of bud-burst; FLD, date of first leaf unfolding; 50LD, date of 50% of leaf unfolding; FFD, first flowering day; PFD, peak flowering day; EFD, end of flowering day. Numbers in brackets indicate the squared semi-partial correlation coefficients. N indicates the number of phenological records.*indicates p < 0.05; **indicates p < 0.01; and ***indicates p < 0.001.

The impact of geographic factors on phenophases varied (Table 2). BBD, FLD and 50LD were delayed in *O. fragrans* by 3.98, 3.93 and 4.40 days on average per degree of latitude, respectively. The geographical factors explained more than 60% of the spatial variation in the spring phenology. No significant relationship was found between leaf phenophases and longitude or altitude, except for the correlation between 50LD and altitude (*p* < 0.01) (Table 2), where 50LD was delayed 0.018 days per m increasing in altitude.

**FIGURE 4:**
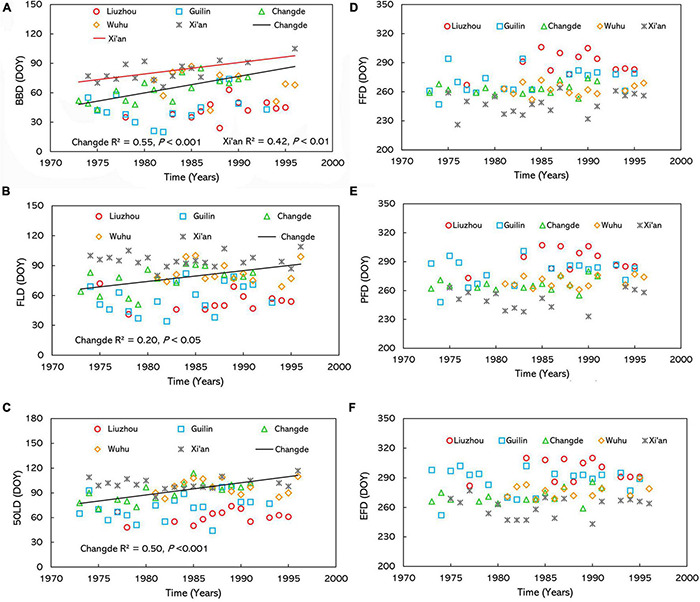
Interannual variations of **(A)** BBD, **(B)** FLD, **(C)** 50LD, **(D)** FFD, **(E)** PFD, and **(F)** EFD for *O. fragrans* at the five observation sites and the mean observation of five sites from 1973 to 1996. BBD, date of bud-burst; FLD, date of first leaf unfolding; 50LD, date of 50% of leaf unfolding; FFD, first flowering day; PFD, peak flowering day; EFD, end of flowering day. DOY indicates the Julian day of year.

Across all the stations, the multiple stepwise regression analysis shows that BBD was delayed by an average of about 0.69 day per year from 1973 to 1996 (*P* < 0.01) (Table 2), whereas FLD and 50LD didn’t show significant temporal trends (Table 2). The data from the stations at Changde and Xi’an had significant positive trends in BBD, but none of other stations (Figure 4A). In addition, the only delayed trends were observed at Changde station in FLD and 50LD (Figures 4B,C and Supplementary Data 8).

### Spatiotemporal Variation of Autumn Flowering Phenophases in *Osmanthus fragrans*

In contrast to spring leaf phenophases, autumn FFD, PFD, and EFD in *O. fragran*s had significant and negative correlations with latitude across the whole area from 1973 to 1996 (Figures 3D–F and Supplementary Data 7). FFD, PFD, and EFD in *O. fragrans* advanced 3.11, 3.26, and 2.99 days, respectively, by every degree northward. In each of these measures, the geographical factors accounted for 61, 61, and 55% of the spatial variation, respectively. There was no significant variation among autumn flowering phenophases regarding longitude and altitude (Table 2 and Supplementary Data 6).

The flowering phenophases of *O. fragrans* showed a delayed trend in the study area (Table 2). FFD was delayed by 0.67 day (*P* < 0.001) (Table 2) per year, PFD was delayed by 0.52 day (*P* < 0.01) per year (Table 2), and EFD was delayed by 0.43 day (*P* < 0.05) (Table 2) per year. However, no significant temporal trends were observed in flowering phenophases in each station (Figures 4D–F and Supplementary Data 8).

### Effects of Climatic Factors on *Osmanthus fragrans* Phenology

**FIGURE 5:**
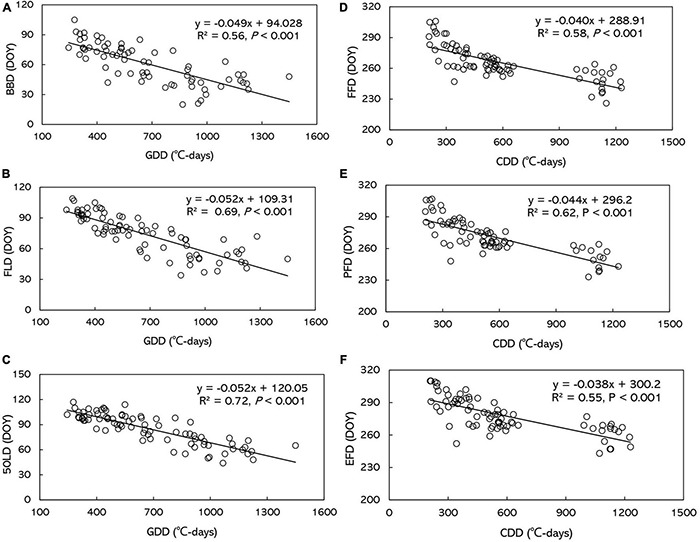
The relationship between the dates of phenophases and growing degree-days/cold degree-days (GDD/CDD) across all stations and years for **(A)** BBD, **(B)** FLD, **(C)** 50LD, **(D)** FFD, **(E)** PFD, and **(F)** EFD. BBD, date of bud-burst; FLD, date of first leaf unfolding; 50LD, date of 50% of leaf unfolding; FFD, first flowering day; PFD, peak flowering day; EFD, end of flowering day. DOY indicates the Julian day of year.

Spring phenophases showed negative correlation with GDD and PPT, and positive correlation with SSD by simple linear regression analyses. However, autumn flowering phenophases were only negatively correlated with CCD, but not with PPT and SSD (Supplementary Table 3 and Supplementary Data 9). SSD was no significantly different during CCD among the five stations by one-way ANOVA (*P* > 0.05). Stepwise multiple linear regression analysis demonstrated that GDD and CDD were the major factors influencing *O. fragrans* phenophases (Table 3). No significant correlations were found between other phenophases and PPT. Only BBD (*P* < 0.01) and FLD (*P* < 0.01) were significantly negatively correlated with PPT. The correlation was extremely weak between phenophases and SSD (Table 3 and Supplementary Data 10). BBD, FLD and 50LD advanced 4.9, 5.2, and 5.2 days, respectively, with every GDD increase of 100°C-days (Figures 5A–C). FFD, PFD and 50LD advanced 4.0, 4.4, and 3.8 days, respectively, with every CDD increase of 100°C-days (Figures 5D–F and Supplementary Data 10).

**TABLE 3:** Partial correlation coefficients between the phenological metrics and climatic factors across all stations and years.

Phenological metric	GDD	CDD	PPT	SSD	R^2^
BBD	−0.64***	/	−0.35**	0.06	0.61***
FLD	−0.75***	/	−0.27**	–0.06	0.71***
50LD	−0.85***	/	0.04	–0.08	0.72***
EFD	/	−0.77***	–0.18	0.02	0.59***
PFD	/	−0.79***	–0.04	0.03	0.62***
EFD	/	−0.77***	0.00	0.14	0.58***

BBD, date of bud-burst; FLD, date of first leaf unfolding; 50LD, date of 50% of leaf unfolding; FFD, first flowering day; PFD, peak flowering day; EFD, end of flowering day. GDD, CDD, PPT, and SSD indicate the growing degree-days, cold degree-days, cumulative precipitation and cumulative sunshine duration, respectively. All climatic factors (GDD, CDD, PPT, and SSD) for BBD, FLD, 50LD, FFD, PFD, and EFD were calculated based on daily meteorological data from 1st January to 30th April and from 1st July to 31^st^ October, respectively. ** indicates p < 0.01; and *** indicates p < 0.001.

### Relationship Between Spring Phenology and Autumn Phenology

**FIGURE 6:**
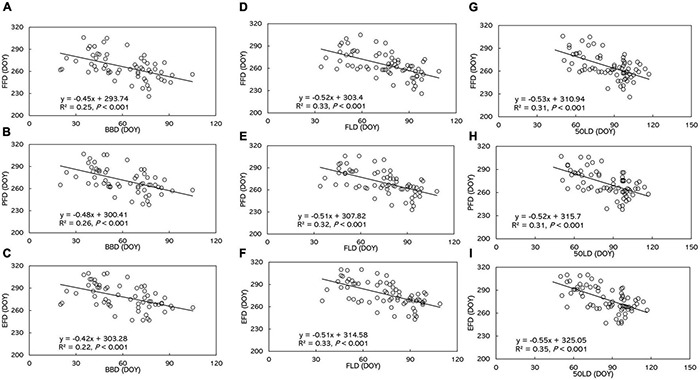
The relationship of **(A)** FFD with BBD, **(B)** PFD with BBD, **(C)** EFD with BBD, **(D)** FFD with FLD, **(E)** PFD with FLD, **(F)** EFD with FLD, **(G)** FFD with 50FLD, **(H)** PFD with 50FLD, **(I)** EFD with 50FLD. BBD, date of bud-burst; FLD, date of first leaf unfolding; 50LD, date of 50% of leaf unfolding; FFD, first flowering day; PFD, peak flowering day; EFD, end of flowering day. DOY indicates the Julian day of year. Numbers in brackets indicate the squared correlation coefficients.

A significantly negative correlation between leaf and flowering phenophases dominated the entire study area from 1973 to 1996 (*R^2^* = 0.22 to 0.35, *P* < 0.001, Figure 6 and Supplementary Data 11). Flowering phenophases were delayed by 0.42–0.48 day by every single day of advanced BBD. FFD, PFD, and EFD were delayed 0.52, 0.51, and 0.51 day, respectively, by every day of advanced FLD. Autumn flowering phenophases were delayed 0.52–0.55 day for every day of advanced 50LD.

## Discussion

### The Effect of Geographical Factors on the Spatial Distributions of Phenology

In this study, there was a clear pattern of the geographical distribution of *O. fragrans* phenology in China. All spring and autumn phenological metrics of *O. fragrans* showed strong dependence on geographical factors (Table 2). We found that spring phenophases were delayed with increased latitude, from south to north, while autumn flowering phenophases were advanced at higher latitudes. In other words, the period between leaf phenology and flowering phenology shortened from south to north (Figure 3). In line with the previous study, we focused on the geographical distribution of leaf phenophases in China (Dai et al., 2014). The spring phenophases were delayed about 4 days per degree of latitude across the studied areas (*P* < 0.001), which is consistent with hypothesis that spring and summer phenologies are delayed 4 days per degree in latitude (Hopkins, 1918; Richardson et al., 2019). These latitudinal patterns are consistent with the previous studies that had examined widespread spring species along latitudinal gradients (Dai et al., 2014; Wang et al., 2015; Gerst et al., 2016; Song et al., 2021). Similar latitudinal patterns were also detected in autumn phenology (Gill et al., 2015; Zhu et al., 2018). Many spring plant phenological events require a certain amount of chilling accumulation for dormancy release (Cook et al., 2012; Fu et al., 2014; Du et al., 2019). Chilling requirements vary among populations of the same species, depending on latitude. Low-latitude populations may require less chilling than that of high-latitude populations (Harrington et al., 2010; Liang, 2016). This latitudinal pattern might be attributed to the long-term adaptation to local climate (Vitasse et al., 2018), and it is likely connected to geographic variation in population genetics (Liang, 2016).

### Changes in Phenology of *Osmanthus fragrans*

This study demonstrated that BBD was significantly delayed at an average rate of 0.69 day per year, while FLD and 50LD displayed marginal trends (Table 2). These findings are inconsistent with most of previous studies (Ge et al., 2015; Wang et al., 2018), which have demonstrated that spring plant phenophases have occurred earlier over the past several decades with global warming. These discrepancies are likely attributed to climate zone. The advance has largely been observed in temperate, boreal, alpine, or in subalpine climates (Shen et al., 2014; Wang et al., 2015). Notably, some studies indeed shown delayed spring bud burst due to climate warming in some warm temperate to subtropical regions (Yu et al., 2010; Zhang et al., 2021), consistent with our finding of spring phenophases. The delay might be due to the warm living habits and the distributional ranges of *O. fragrans*. The insufficient accumulation of chilling temperature during dormancy counteracted the force of temperature accumulation induced by climate warming, which in total delayed leaf phenophases (Xie et al., 2015; Chen et al., 2017). Meanwhile we found that autumn flowering phenophases were significantly delayed in *O. fragrans* (Table 2), which is consistent with the patterns in other autumn phenological studies (Ge et al., 2015; Shen et al., 2020). Previous reports on fall phenophases, such as leaf senescence and the end of season (EOS), have revealed substantially delayed trends associated with increasing temperatures (Gallinat et al., 2015; Cong et al., 2017; Fu et al., 2017). Control and observation experiments have also shown that the flowering of fall flowering species is often delayed (Sherry et al., 2007; Pearson, 2019). The delays in reproductive phases generally could be attributed to the fact that warming delays the fulfillment of chilling requirements and thus leads to later reproduction (Yu et al., 2010; Peng et al., 2019).

In addition, different temporal trends in *O. fragrans* phenology have been found at each site, suggesting that plant phenology may also be influenced by other complex factors. In addition to temperature, other environmental factors such as precipitation, photoperiod and nutrient availability, as well as biological factors, may have certain impacts on spring and autumn phenology (Piao et al., 2019). Photoperiod and temperature may co-regulate plant phenology (Fu et al., 2020), and the effect of precipitation on plant phenology can be explained by its indirect impacts on the thermal requirement (GDD or CDD) for both spring and autumn phenological events (Fu et al., 2014; Hänninen, 2016). Further, the interplay between soil water content and nutrients also has been linked to plant phenology (Estiarte and Peñuelas, 2015). Our results reinforce the need for phenological analysis of the interaction of multiple environmental factors. In order to fully understand the physiological and molecular genetic mechanisms underlying the phenological patterns and processes, controlled physiological experiments are needed.

### Climatic Factors Driving the Spatiotemporal Variation in Phenology

Previous studies have shown that the spatiotemporal variation of plant phenology was strongly driven by climate, and that temperature was the primary factor influencing plant phenology (Jeong et al., 2011; Xu and Chen, 2013). This study observed significant negative partial correlations between spring/autumn phenophases and GDD/CDD in *O. fragrans* across all stations and years. More thermal accumulation tended to advance the occurrence of spring phenophases, while less heat deficit resulted in delayed autumn phenophases (Figure 5 and Table 3). Similarly, negative relationships have been discovered between temperature and phenological events, such as flowering and leaf-out (Willis et al., 2017). The relationships between plant autumn phenology and various climatic factors are more complicated (Ge et al., 2015; An et al., 2020). Temperature, expressed in terms of CDD, was supposed to be the most important controlling factor during the end of season (EOS) in deciduous forests (Dragoni and Rahman, 2012). Air temperature and leaf senescence have been linked at the bio-molecular scale (Allona et al., 2008). Previous studies have reported that cumulative cold temperatures below a certain threshold trigger autumn phenology (Delpierre et al., 2009; Dragoni and Rahman, 2012; Gill et al., 2015). Warmer temperatures in autumn lower CDD and postpone autumn phenology (Ge et al., 2015; Liu et al., 2016b; Shen et al., 2020). For autumn flowering species, flowering dates were delayed by warm preseason temperature (Sherry et al., 2007; Pearson, 2019), most of which were consistent with our studies.

Our results showed that BBD and FLD advanced with increased precipitation, while other phenological metrics remain unchanged (Table 3), which are consistent with those reported by previous studies (Hänel and Tielbörger, 2015; Zhou et al., 2019). Water availability played a dominant role in regulating plant growth (Liu et al., 2009). In other words, plants grow earlier with increased precipitation, accordingly, increased soil water content. In addition, the temporal distribution of precipitation, rather than the total amount, may be more related to plant phenology (Dragoni and Rahman, 2012).

In our study, the spring phenology of *O. fragrans* was positively correlated with SSD by simple linear regression (Supplementary Table 3), but weak relationship between spring phenology and SSD by stepwise multiple linear regression analysis (Table 3). Our results are consistent with these previous studies (Polgar et al., 2014; Du et al., 2019; Song et al., 2020b). For all species, the responses of phenology to photoperiod are species-specific (Basler and Körner, 2014). Most woody plant species are not sensitive to photoperiod, even for the photoperiod sensitive species, many studies suggest their budburst dates were not sensitive to photoperiod, when the chilling demand was met (Zohner et al., 2016).

The effect of one climatic factor could be exacerbated or mitigated by another (Mitchell et al., 2015), so accurate prediction of climate change on plant phenology requires a holistic consideration of the joint and interactive effects of all factors. However, few experimental studies have quantified the individual and combined effects of different environmental cues on plant phenology (Pérez-Ramos et al., 2020).

## Conclusion

In our study, we demonstrated that the spring phenophases of *O. fragrans* were delayed and the autumn phenophases were advanced as latitude increased. Further analysis found that temperature rather than precipitation was the most important driver of the spatiotemporal variation in the phenologies of *O. fragrans*. In addition to the effects of climate on autumn flowering phenology, earlier spring leaf phenophases could cause delayed autumn flowering phenophases. Overall, these results suggest that temperature affects the autumn phenology of *O. fragrans* through both direct and indirect factors. Therefore, the future climate warming may further delay the autumn phenological sequence of *O. fragrans*, thus, it is necessary to conduct deeper studies of the autumn phenology of *O. fragrans*.

## Data Availability Statement

The raw data supporting the conclusions of this article will be made available by the authors, without undue reservation.

## Author Contributions

XW and FS conceived the ideas. XW, YL, XL, and MZ analyzed the data. XW, XL, and SH drew Figure. XW drafted the manuscript. All authors contributed to the article and approved the submitted version.

## Conflict of Interest

The authors declare that the research was conducted in the absence of any commercial or financial relationships that could be construed as a potential conflict of interest.

## Publisher’s Note

All claims expressed in this article are solely those of the authors and do not necessarily represent those of their affiliated organizations, or those of the publisher, the editors and the reviewers. Any product that may be evaluated in this article, or claim that may be made by its manufacturer, is not guaranteed or endorsed by the publisher.

## Footnotes

1
http://data.cma.cn/
