# Supplementary material for: Spatiotemporal Variation of Osmanthus fragrans Phenology in China in Response to Climate Change From 1973 to 1996
Source: Front Plant Sci. 2022 Jan 20;12:716071. doi: 10.3389/fpls.2021.716071 (PMC8811162; doi:10.3389/fpls.2021.716071)
Supplement: Supplementary file 1 [file Table_1.DOCX]

Table S1: Partial correlation coefficient of phenological metrices with climatic factors using 0 ℃ and 10℃ as GDD temperature threshold.

| Base temperatures | Phenological  metric | GDD | PPT | SSD | R2 |
| --- | --- | --- | --- | --- | --- |
| 0 | BBD | -0.65*** | -0.25* | -0.007 | 0.62*** |
|  | FLD | -0.85*** | -0.14 | -0.05 | 0.73*** |
|  | 50LD | -0.84*** | 0.16 | -0.21 | 0.70*** |
| 10 | BBD | -0.36** | -0.21 | 0.41** | 0.55*** |
|  | FLD | -0.69*** | -0.38** | 0.06 | 0.65*** |
|  | 50LD | -0.81*** | -0.12 | 0.14 | 0.65*** |

BBD, date of bud-burst; FLD, date of first leaf unfolding; 50LD, date of 50% of leaf unfolding; FFD, first flowering day; PFD, peak flowering day; EFD, end of flowering day. GDD, PPT and SSD indicate the growing degree-days, accumulated precipitation and sunshine duration, respectively. All climatic factors (GDD, CDD, PPT and SSD) for BBD, FLD, 50LD, FFD, PFD and EFD were calculated based on daily meteorological data from1st January to 30th April. * indicates p < 0.05, ** indicates p < 0.01; and *** indicates p < 0.001.
