# Supplementary material for: Spatiotemporal Variation of Osmanthus fragrans Phenology in China in Response to Climate Change From 1973 to 1996
Source: Front Plant Sci. 2022 Jan 20;12:716071. doi: 10.3389/fpls.2021.716071 (PMC8811162; doi:10.3389/fpls.2021.716071)
Supplement: Supplementary file 2 [file Table_2.DOCX]

Table S2. Partial correlation coefficients of phenological metrices with climatic factors using 20℃ and 30℃ as CDD temperature threshold.

| Base temperatures | Phenological  metric | CDD | PPT | SSD | R2 |
| --- | --- | --- | --- | --- | --- |
| 20 | FFD | -0.70*** | -0.16 | 0.05 | 0.48*** |
|  | PFD | -0.72*** | -0.04 | 0.08 | 0.51*** |
|  | EFD | -0.70*** | 0.00 | 0.16 | 0.49*** |
| 30 | FFD | -0.80*** | -0.12 | -0.02 | 0.63*** |
|  | PFD | -0.81*** | 0.06 | -0.03 | 0.65*** |
|  | EFD | -0.77*** | 0.07 | 0.11 | 0.59*** |

BBD, date of bud-burst; FLD, date of first leaf unfolding; 50LD, date of 50% of leaf unfolding; FFD, first flowering day; PFD, peak flowering day; EFD, end of flowering day. CDD, PPT and SSD indicate cold degree-days, accumulated precipitation and sunshine duration, respectively. All climatic factors ( CDD, PPT and SSD) for FFD, PFD and EFD were calculated based on daily meteorological data from 1st August to 31^st^ October. * indicates p < 0.05, ** indicates p < 0.01, and *** indicates p < 0.001.
