# Supplementary material for: Spatiotemporal Variation of Osmanthus fragrans Phenology in China in Response to Climate Change From 1973 to 1996
Source: Front Plant Sci. 2022 Jan 20;12:716071. doi: 10.3389/fpls.2021.716071 (PMC8811162; doi:10.3389/fpls.2021.716071)
Supplement: Supplementary file 3 [file Table_3.DOCX]

Table S3. Correlation coefficient of phenological metrices with climatic factors.

|  | GDD | CDD | PPT | SSD |
| --- | --- | --- | --- | --- |
| BBD | -0.75*** |  | -0.60*** | 0.71*** |
| FLD | -0.83*** |  | -0.59*** | 0.71*** |
| 50LD | -0.85*** |  | -0.45*** | 0.65*** |
| FFD |  | -0.77*** | -0.09 | 0.07 |
| PFD |  | -0.79*** | 0.16 | 0.15 |
| EFD |  | -0.77*** | 0.21 | 0.15 |

bud-burst; FLD, date of first leaf unfolding; 50LD, date of 50% of leaf unfolding; FFD, first flowering day; PFD, peak flowering day; EFD, end of flowering day. CDD, PPT and SSD indicate cold degree-days, accumulated precipitation and sunshine duration, respectively. All climatic factors (GDD, PPT and SSD) for BBD, FLD and 50LD were calculated based on daily meteorological data from1st January to 30th April. (CDD, PPT and SSD) for FFD, PFD and EFD were calculated based on daily meteorological data from 1st August to 31st October. * indicates p < 0.05, ** indicates p < 0.01, and *** indicates p < 0.001.
